# Supplementary material for: Stress routes clients to the proteasome via a BAG2 ubiquitin-independent degradation condensate
Source: Nat Commun. 2022 Jun 2;13:3074. doi: 10.1038/s41467-022-30751-4 (PMC9163039; doi:10.1038/s41467-022-30751-4)
Supplement: Supplementary file 1 — Supplementary Information [file 41467_2022_30751_MOESM1_ESM.pdf]

Supplementary Information

"Stress Routes Clients to the Proteasome via a BAG2 Ubiquitin-Independent Degradation Condensate"

Daniel C. Carrettiero[1,2], Maria C. Almeida[1,2], Andrew P. Longhini[1], Jennifer N. Rauch[1], Dasol Han[1], Xuemei Zhang[1], Saeed Najafi [3], Jason E. Gestwicki[4], Kenneth S. Kosik[1]*

1. Neuroscience Research Institute, Department of Molecular, Cellular, and Developmental Biology, University of California, Santa Barbara, CA, USA

2. Center for Natural and Humanities Sciences, Federal University of ABC, São Bernardo do Campo, SP, Brazil

3. Department of Chemistry and Biochemistry, University of California, Santa Barbara, CA, USA

4. Institute for Neurodegenerative Disease, Department of Pharmaceutical Chemistry, University of California San Francisco, San Francisco, CA, USA

* Corresponding author, kosik@lifesci.ucsb.edu

Supplementary Figure 1

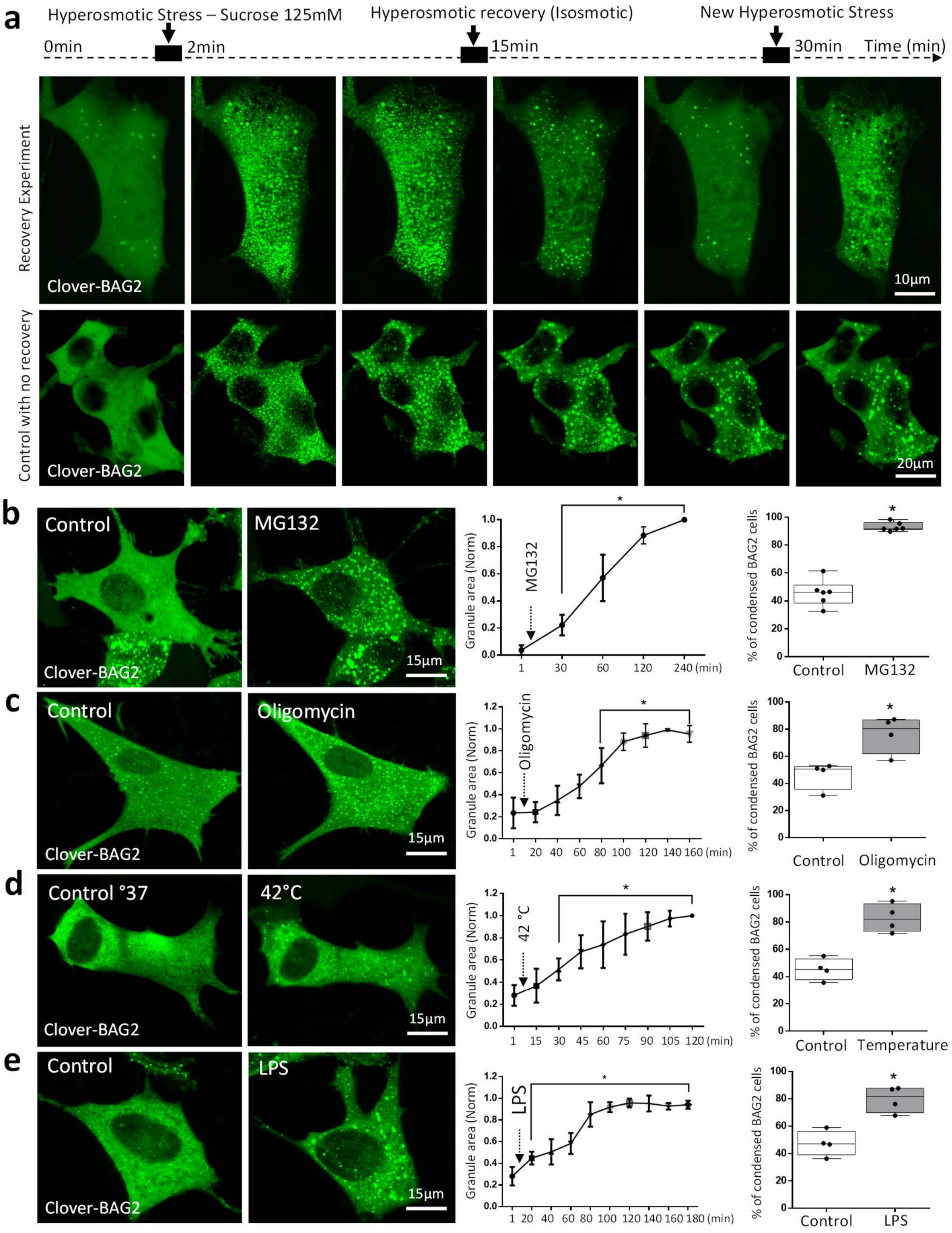

Supplementary Figure 1. BAG2 condensation in response to stress a) Time-lapse images of a cell subjected to hyperosmotic stress (Sucrose, 125mM) at 2min, osmotic recovery at 15 min, and

hyperosmotic stress again at 30 min. Dissolution of BAG2 condensates occurred with recovery from osmotic stress. Osmotic recovery was obtained adding appropriated amount of water to the medium to reach the control osmolarity value. The bottom images show a similar time lapse of cells submitted to hyperosmotic stress but not to osmotic recovery. **b-e)** Representative images and respective quantification of the BAG2 condensate area over time and percentage of cells expressing BAG2 in dense phase after different types of stressors were shown: (b) MG132 (proteasome inhibition, 10 μM, 240 min); (c) Oligomycin (ATP depletion, 1 μM, 160 min; (d) Temperature (42°C, 120 min); (e) Lipopolysaccharide, LPS (Inflammatory signal, 10 μg ml^{-1}, 180 min. Condensation areas are shown as the sum of clover-BAG2 condensation area normalized by its highest value (*p = $2.15x10^{-5}$, 6 cells for MG132; *p = $3.6x10^{-10}$, 3 cells for Oligomycin; *p = $6.3x10^{-6}$, 5 cells for Temperature; *p = $2.3x10^{-6}$, 5 cells for LPS, one-way ANOVA followed by the Dunnett's test , mean ± SD). All data are from 3 independent replicates. The percentage of clover-BAG2 cells that contained condensates were analyzed and plotted (*p = $3.9x10^{-7}$ for MG132, n=6; p = $1.2x10^{-2}$ for Oligomycin, n=4; p = $1.3x10^{-3}$ for Temperature, n=4; p = $2.8x10^{-3}$ for LPS, n=4; 600-1,000 cells per treatment, two-sided Student's t test). Experiments performed in clover-BAG2 stable SH-SY5Y cells. Box and whiskers: the boxplots are centered around the median and extend from the 25th to 75th percentiles. The whiskers go down to the smallest value and up to the largest. Dots represents individual BAG2 condensate.

Supplementary Figure 2

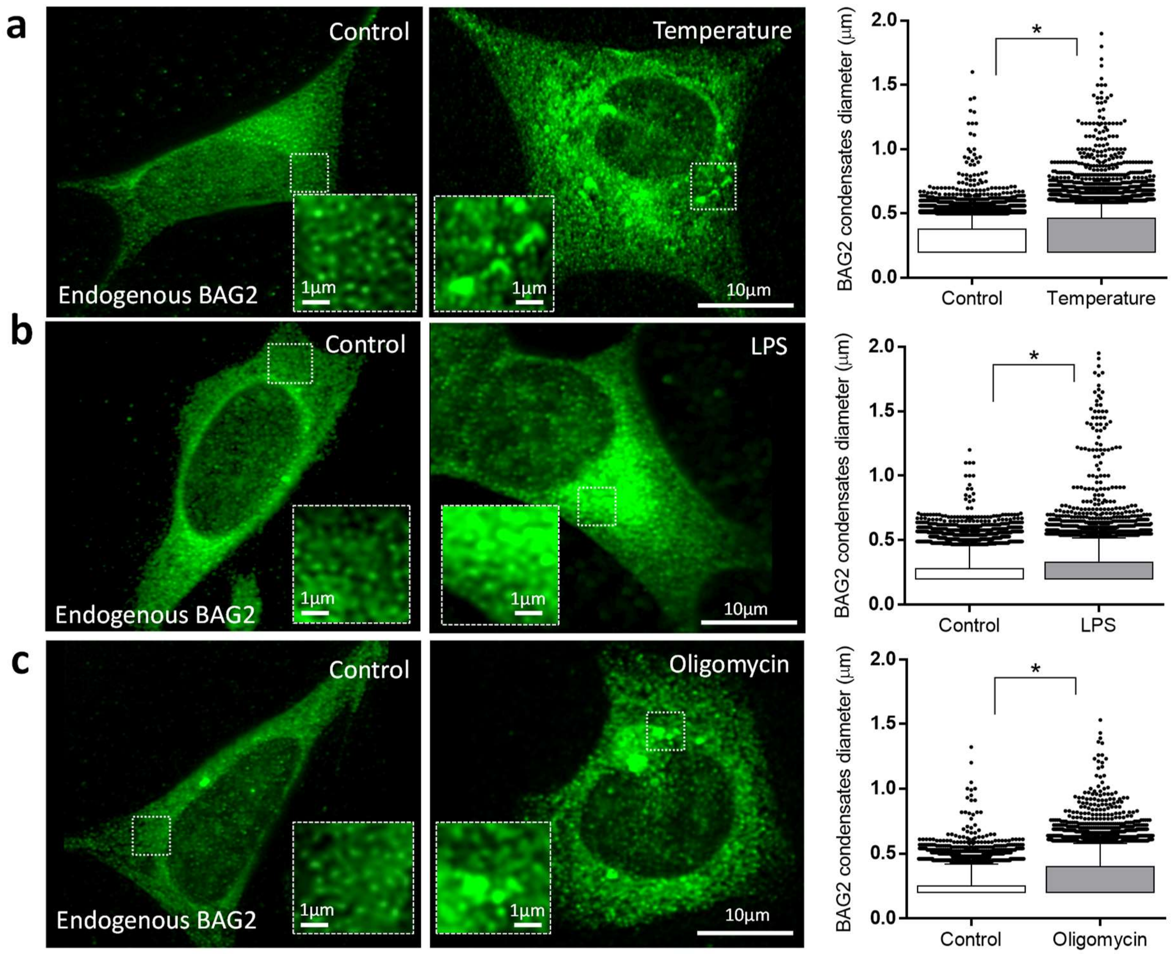

Supplementary Figure 2. Endogenous BAG2 condensation in response to different stresses. Immunostaining images of endogenous BAG2 in SH-SY5Y cells in control and after different stresses **a)** Temperature (42°C, 120 min) increases BAG2 condensates diameter (insert). Graph (*p = 1.7x10^{-100}, two-sided Students' t-test, 12 cells analyzed with a total of 13,017 puncta for control and 11 cells analyzed with a total of 10,752 puncta for 42°C). **b)** lipopolysaccharide (LPS; 180 min) increases BAG2 condensates size (insert). Graph (*p = 4.9x10^{-19}, two-sided Students' t-test, 10 cells analyzed with a total of 6,990 puncta for control and 11 cells analyzed with a total

of 6,556 puncta for LPS). **c)** Oligomycin (120 min) increases BAG2 condensates size (insert). Graph (*p = 9.2×10^{-101}, two-sided Students' t-test, 10 cells analyzed with a total of 7,509 puncta for control and 12 cells analyzed with a total of 6,810 puncta for Oligomycin). **a-c**: Size detection lower than 0.20 µm (about the maximum confocal resolution) were cut off from the analysis. Box and whiskers: the boxplots are centered around the median and extend from the 25th to 75th percentiles. 3 independent replicates. The whiskers are drawn down to the 10th and up to 90th percentile. Points below and above the whiskers are drawn as individual dots. Dots represents individual BAG2 condensate.

Supplementary Figure 3

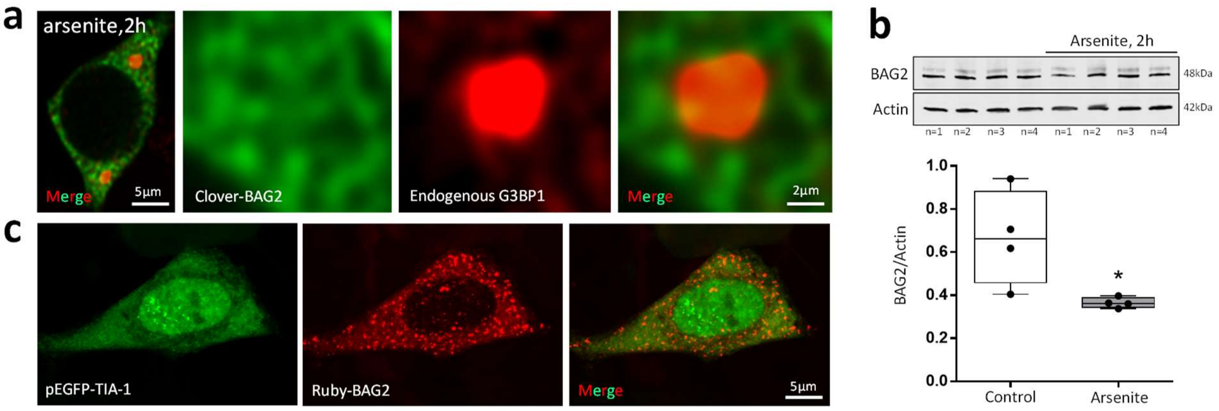

Supplementary Figure 3. BAG2 condensates are distinct from stress granules. a) Representative image of a SH-SY5Y cell stably expressing clover-BAG2 exposed to arsenite (500 µM, 120 min), and immunostained for endogenous G3BP1, a stress granule marker, showing no colocalization between BAG2 and G3BP1 condensate. **b)** Stably expressing BAG2 cells subjected to arsenite treatment (500 µM, 120 min) were resolved by Western blotting against BAG2. Beta-actin was used as a loading and normalization control. The levels of BAG2 decreased after arsenite treatment (*p = 6.0×10^{-4}, two-sided Student's t-test)**.** Representative blots and the densitometry quantification of 4 independent replicates are shown. Box and whiskers: the boxplots are centered around the median and extend from the 25th to 75th percentiles. The whiskers go down to the smallest value and up to the largest. Dots represents replicates. **c)** Representative image of SH-SY5Y cell co-transfected with ruby-BAG2 and pEGFP-TIA1, another stress granule marker, showing no colocalization between BAG2 condensates and TIA1. 3 independent replicates.

Supplementary Figure 4

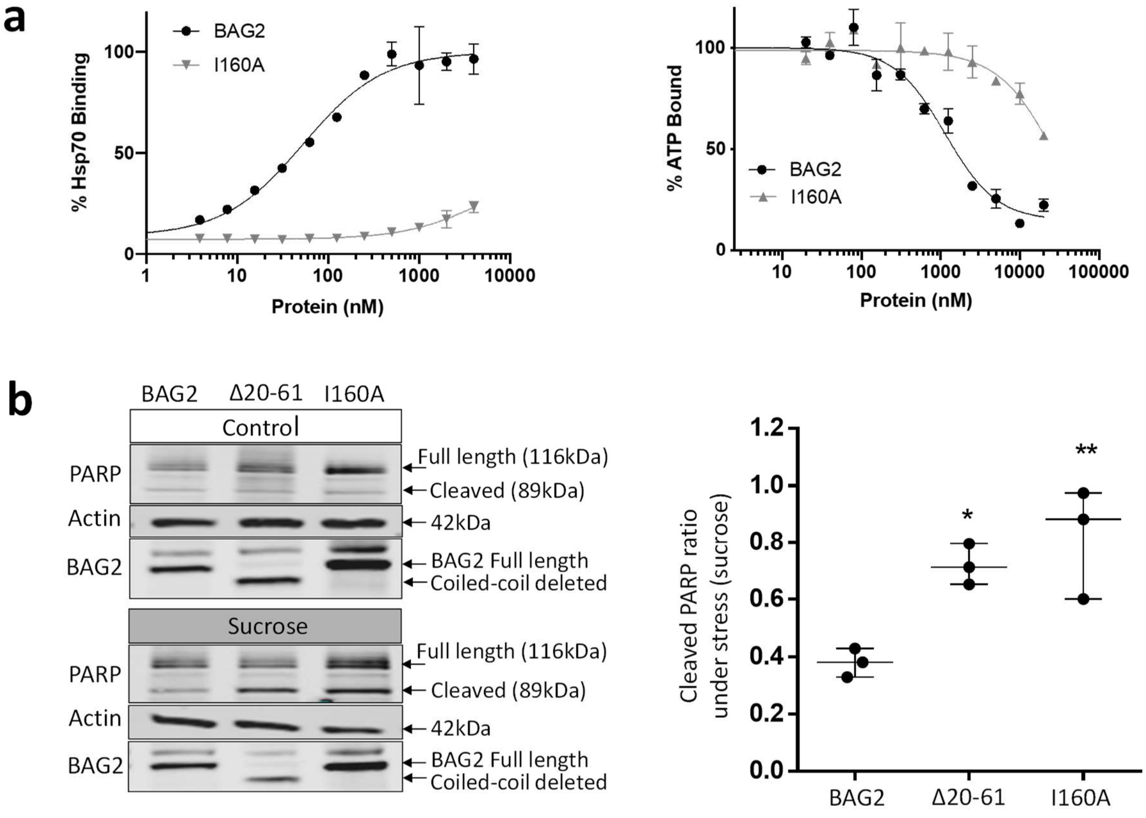

Supplementary Figure 4. Structural determinants of BAG2 condensates and cell protection. a) Percentage of Hsp70 binding measured by flow cytometry protein interaction assay (FCPIA, left) and nucleotide release from Hsp70 measured by fluorescence polarization (FP, right) by BAG2I160A. Experiments were performed in triplicate, mean ± SD. **b)** Stably expressing BAG2 full length, BAG2Δ20-61 and BAG2I160A SH-SY5Y cells subjected to hyperosmotic stress (500 mM, 120 min) were resolved by Western blotting against PARP and BAG2. Beta-actin was used as a loading control. Levels of cleaved PARP ratio were increase in both mutants after stress, as compared to BAG2 full length. *p = $2.0x10^{-2}$, **p = $8.0x10^{-3}$, one-way ANOVA followed by the Tukey`s post-test. Representative blots and the densitometry quantification of 3 independent

replicates are shown. The lines are centered around the median. Plots are drown from min. to max. values. Dots represent replicates.

Supplementary Figure 5

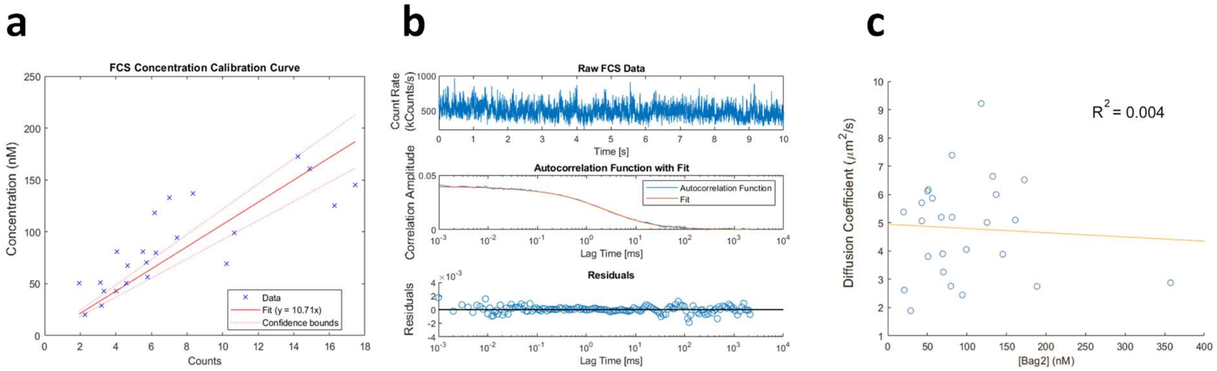

Supplementary Figure 5. Fluorescence correlation spectroscopy (FCS). Representative measured FCS traces used to calculate the concentration of BAG2. **a)** Concentrations as determined from FCS measurements were correlated with the intensity counts at the same location. The data were fit to a linear model and used to calculate the concentrations of the BAG2 dilute and dense phases. **b)** Raw FCS Data: Raw photon counts measured over 30 seconds. Autocorrelation function with fit: The fit autocorrelation function of the raw photon counts. Fitting used a Triplet Extended 3D model as described in the methods. Residuals: Residuals for the fitted autocorrelation function. **c)** Plot of the fitted concentrations of BAG2 calibration samples versus the diffusion coefficients as measured by FCS. A range of diffusion coefficients indicate the transient interactions with different diffusion times are present in the samples. The data were fit to a simple linear model and an R2 of 0.004 confirmed that little to no correlation was seen between the concentration and the diffusion coefficient. The lack of a correlation between the concentration of molecules and the diffusion coefficient help to support those fluctuations are due to heterotypic interactions and not homotypic interactions.

Supplementary Figure 6

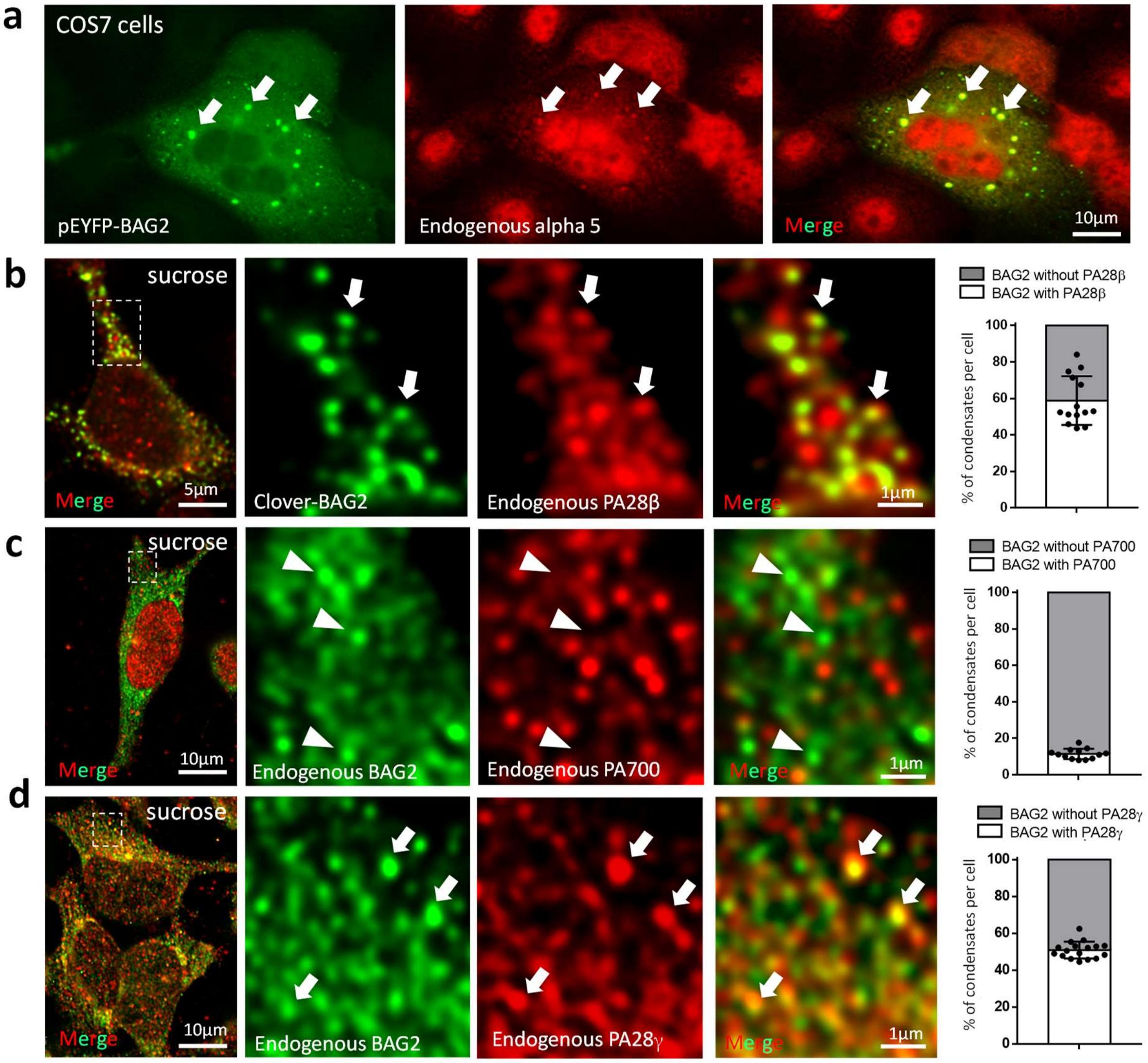

Supplementary Figure 6. BAG2 condensates recruits 20S proteasomes and the proteasome activator PA28. a) Representative images of COS7 cells transfected with pEYFP-BAG2 and immunostained for endogenous alpha 5 subunit of 20S proteasome. Arrow shows colocalization between BAG2 condensates and alpha 5 subunit. **b)** Images showing stably expressing clover-BAG2 SH-SY5Y cells exposed to hyperosmotic stress (Sucrose, 125mM, 30 min), fixed and stained for endogenous PA28 Beta. Arrows represent co-localization between BAG2 and PA28

Beta. The plot shows co-localization quantification (1,828 condensates analyzed in 14 cells from 3 independent replicates). **c)** Immunostaining images of endogenous BAG2 and endogenous PA700 (19S cap) in SH-SY5Y exposed to hyperosmotic stress (Sucrose, 125mM, 30 min). Arrowheads represent no co-localization between BAG2 and PA700. The plot shows co-localization quantification (6,207 condensates analyzed in 13 cells from 3 independent replicates). **d)** Immunostaining images of endogenous BAG2 and endogenous PA28 gamma in SH-SY5Y exposed to hyperosmotic stress (Sucrose, 125mM, 30 min). Arrows represent co-localization between BAG2 and PA28 gamma. The plot shows co-localization quantification (6,990 condensates analyzed in 17 cells from 3 independent replicates). **b-d**, Dots represent the percentage of BAG2 condensates colocalized with PA28 beta, PA700 (19S cap) and PA28 gamma from each individual cell. Line is centered around the mean ± SD.

Supplementary Figure 7

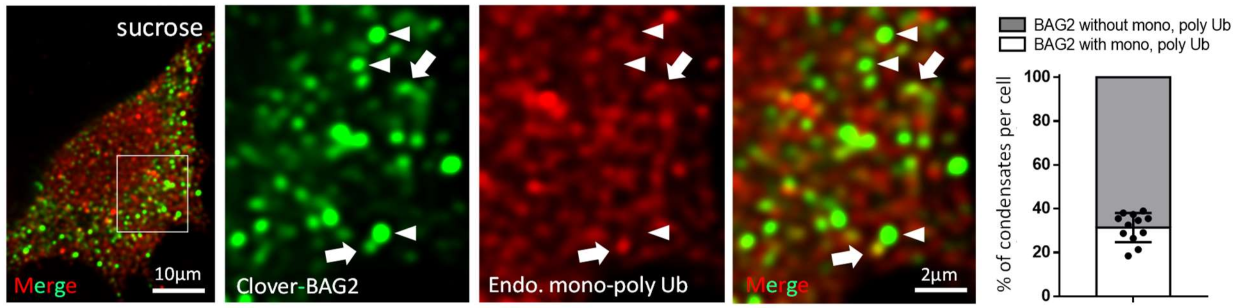

Supplementary Figure 7. BAG2 condensates function as degradation granule. Images showing stably expressing clover-BAG2 SH-SY5Y cells exposed to hyperosmotic stress (Sucrose, 125mM, 30 min), fixed and stained for endogenous mono- and poly-ubiquitinylated conjugates. In most BAG2 condensates ubiquitin immunolabeling was absent (arrowhead). Arrow shows colocalization between BAG2 condensates and ubiquitin immunolabeling. The plot shows co-localization quantification (1,891 condensates, 12 cells, 3 independent replicates). Dots represent the percentage of BAG2 condensates colocalized from each individual cell. Line is centered around the mean ± SD.

Supplementary Figure 8

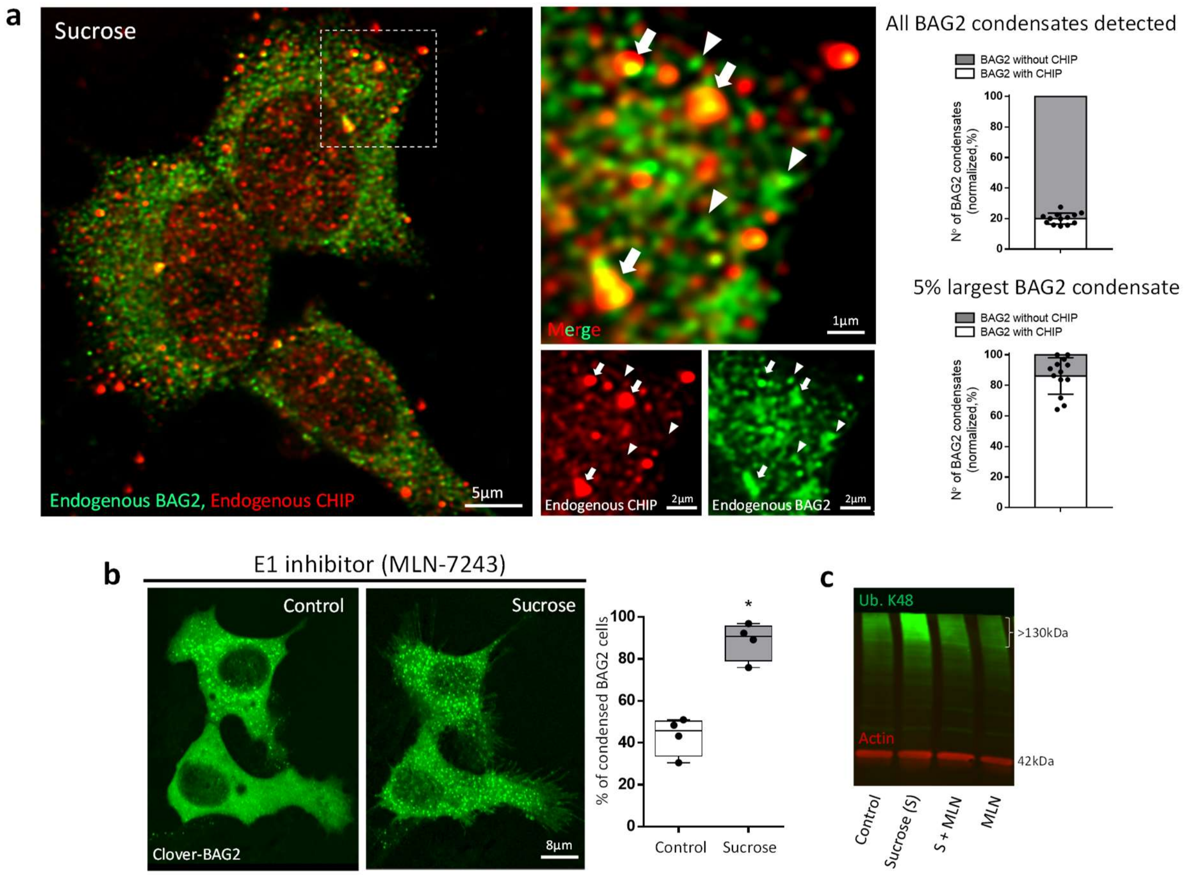

Supplementary Figure 8. BAG2-mediated degradation pathway (ubiquitin independent) vs CHIP-mediated (ubiquitin dependent) one. a) Immunostaining images of endogenous BAG2 and CHIP in SH-SY5Y exposed to hyperosmotic stress (Sucrose, 125mM, 30 min). Only a small percentage of the whole BAG2 condensates population colocalize with CHIP (~20%, arrow). The upper plot shows co-localization quantification (7,479 condensates analyzed in 13 cells from 3 independent replicates). However, a high colocalization (~80% arrowhead) of BAG2 with CHIP was observed when 5% of the largest BAG2 condensates was selected from the total pool. The bottom plot shows co-localization quantification between 280 largest BAG2 condensates and CHIP. Dots represent the percentage of BAG2 condensates colocalized with CHIP from each

individual cell. 13 cells from 3 independent replicates. Line is centered around the mean ± SD. Mean size of 5% largest BAG2 condensates analyzed: 0.75 ± 0.08 µm, SD (graph not shown). **b)** Stably expressing clover-BAG2 SH-SY5Y cells were pre-treated with the ubiquitin E1 inhibitor MLN-7243 (1 µM, 1h) and imaged before and after hyperosmotic stress exposure (125mM of Sucrose, 15 min). MLN-7243 failed to block the formation of BAG condensates. Graphical representation of the percentage of clover-BAG2-positive cells that contained condensates before and after hyperosmotic stress (sucrose) (*$p = 4x10^{-4}$, two-sided Student's t-test, 899 cells for control, 846 cells for hyperosmotic stress, 4 independent replicates). Box and whiskers: the boxplots are centered around the median and extend from the 25th to 75th percentiles. The whiskers go down to the smallest value and up to the largest. Dots represents replicates. **c)** Stably expressing BAG2 full length SH-SY5Y cells subjected to hyperosmotic stress (500 mM, 30 min) and MLN-7243 were resolved by Western blotting against Ubiquitin K48. Beta-actin was used as a loading control. MLN-7243 blocked the high molecular height species formed by ubiquitin-K48 target linkage after hyperosmotic stress. 3 independent replicates.

Supplementary Figure 9

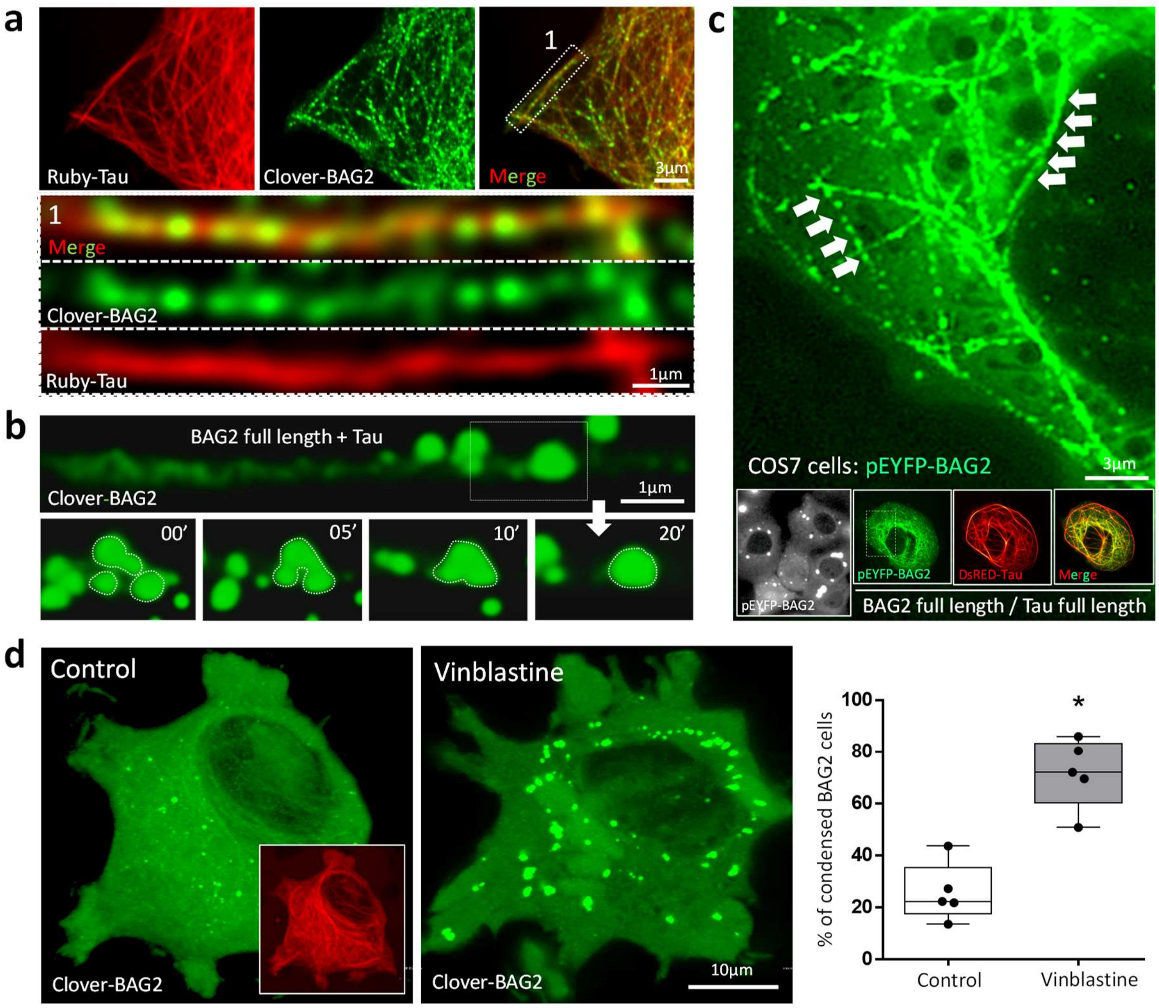

Supplementary Figure 9. BAG2 condensates align along the microtubule with high levels of Tau. a) Representative images of SH-SY5Y cells co-transfected with clover-BAG2 full length and ruby-Tau showing alignment of BAG2 condensates along the microtubule (1). 6 independent replicates. **b)** Representative images of BAG2 condensates showing fusion of condensates on the microtubule over time. 3 independent replicates. **c)** Images showing BAG2 condensates alignment along the microtubule (arrows) in COS-7 cells co-transfected with pEGFP-BAG2 full length and DsRED-Tau. Gray image shows a COS-7 cell transfected only with pEGFP-BAG2. 3

independent replicates. **d)** Representative images of stably expressing clover-BAG2 and ruby-Tau SH-SY5Y cells exposed to vinblastine (5 µM), a microtubule depolymerizing agent for 60min (Supplementary Movie 5). The percentage of clover-BAG2-positive cells that containing condensates increase in response vinblastine as shown in the plot (546 cell for control and 552 cells for Vinblastine, 5 independent replicates, $*p = 4.0 \times 10^{-4}$, two-sided Student's t-test). Box and whiskers: the boxplots are centered around the median and extend from the 25th to 75th percentiles. The whiskers go down to the smallest value and up to the largest. Dots represents replicates.

Supplementary Figure 10

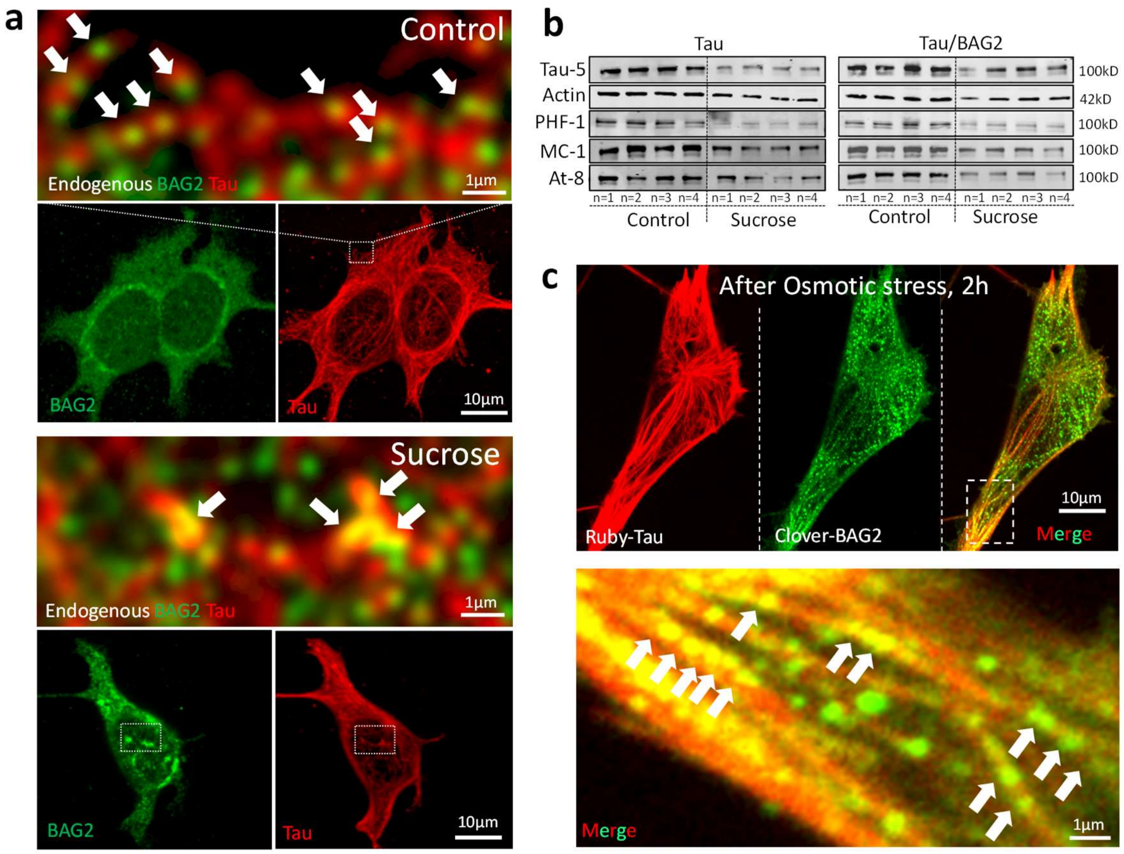

Supplementary Figure 10. BAG2 condensates align along the microtubule and degrade Tau after hyperosmotic stress. a) Representative images of SH-SY5Y cells immunolabeled for endogenous BAG2 and endogenous Tau revealing association between BAG2 condensates and Tau staining on the microtubules in control (top panels, arrows), and brighter and bigger clusters of BAG2 staining co-localizing with regions containing dense Tau staining (bottom panels, arrows) after hyperosmotic stress (Sucrose, 125mM, 30 min). 3 independent replicates.

b) Representative blots of the quantitative results of main Figure 6e are shown. Stable cells expressing either Tau or Tau-P2A-BAG2 were subjected to hyperosmotic stress (sucrose 500 mM, 120 min) and resolved by western blotting with the antibodies Tau-5, PHF-1, MC-1, and AT-8. The samples derive from the same experiment and blots were processed in parallel for Tau or Tau-P2A-BAG2. **c)** Representative images of a stably expressing clover-BAG2-P2A-ruby-Tau SH-SY5Y cell after hyperosmotic stress (500 mM sucrose, 2h) showing BAG2 condensates still aligned along the microtubule 2h after hyperosmotic stress (arrows). 3 independent replicates.

Supplementary Figure 11

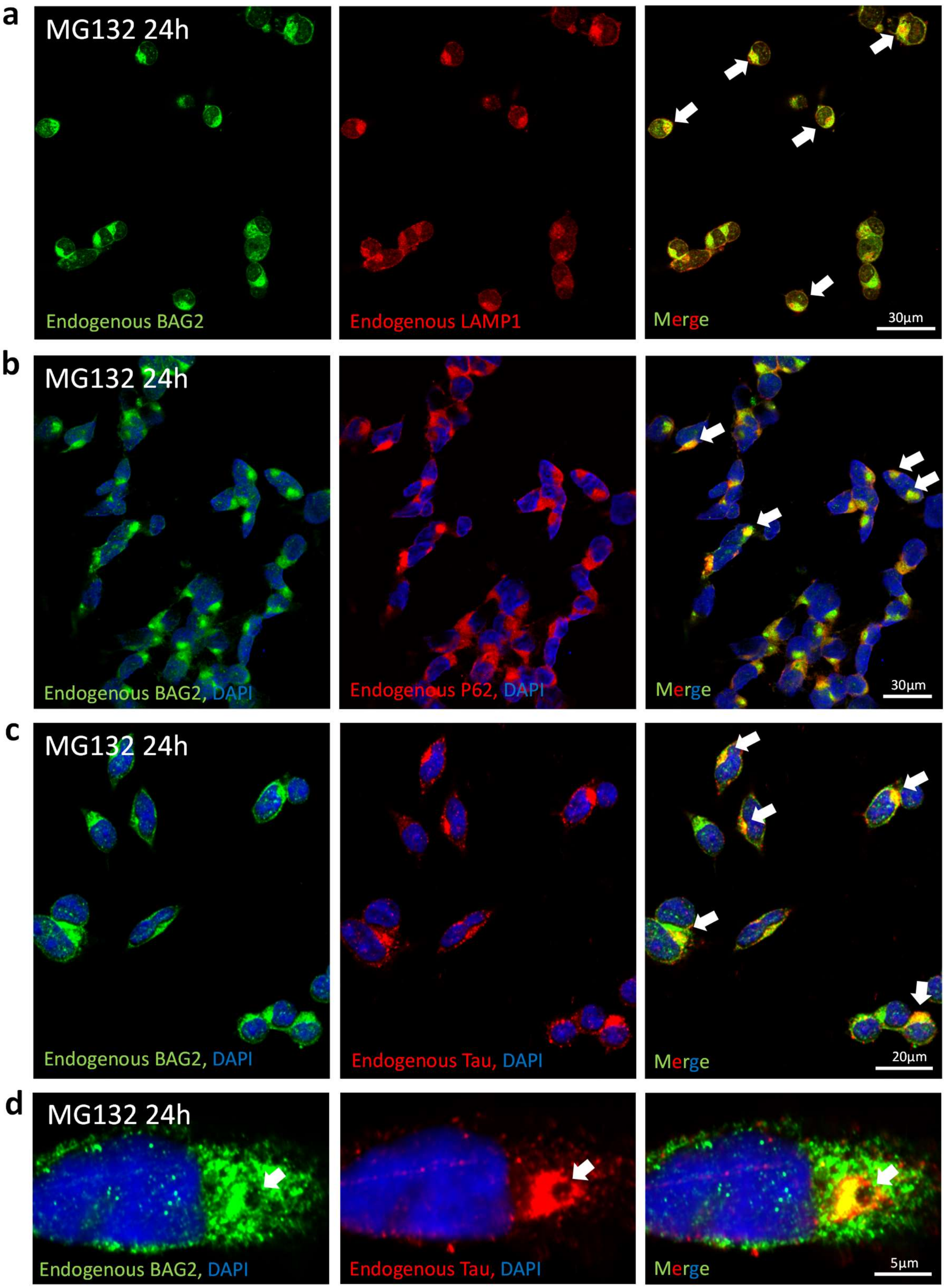

Supplementary Figure 11. BAG2 condensates are directed toward autophagy under UPS inhibition. a-c) Co-immunostaning images of endogenous BAG2 and endogenous LAMP1 (a), endogenous p62/SQSTM1 (b), and endogenous Tau (c) in SH-SY5Y cells treated with MG132 (24h, 10 µM). The images show a well-defined structure in the perinuclear region of the cell containing BAG2 and the proteins LAMP1 (a), p62/SQSTM1 (b) and Tau (c) (arrows) along several cells in low magnification. 3 independent replicates. **d)** The well-defined BAG2-negative core previously described in the main Figure 7f, also presented no endogenous Tau label (arrow) in SH-SY5Y cells treated with MG132 for 24h.
