# Supplementary material for: Stress routes clients to the proteasome via a BAG2 ubiquitin-independent degradation condensate
Source: Nat Commun. 2022 Jun 2;13:3074. doi: 10.1038/s41467-022-30751-4 (PMC9163039; doi:10.1038/s41467-022-30751-4)
Supplement: Supplementary file 3 — Description of Additional Supplementary Files [file 41467_2022_30751_MOESM3_ESM.pdf]

Description of Additional Supplementary Files

File Name: Supplementary Movie 1
Description: BAG2 condensates in stable SH-SY5Y cells. This video shows fusion of clover-BAG2 granules over time. Image-deconvolution algorithms were applied using the Huygens Essential software followed by blend mode in Imaris software (Opacity applied to voxels – 3D impression).

File Name: Supplementary Movie 2
Description: Hyperosmotic stress induces BAG2 condensates in stable SH-SY5Y cells. This video shows clover-BAG2 granules formation before (first frame) and after Stress (Sucrose, 125mM) over time.

File Name: Supplementary Movie 3
Description: BAG2 condensates under Sodium arsenite treatment in stable SH-SY5Y cells. This video shows clover-BAG2 granules clearance after sodium arsenite exposure, a known SG inducer (NaAsO2, 500 µM) over time.

File Name: Supplementary Movie 4
Description: BAG2 condensates and Tau on the microtubules in transient transfected SH-SY5Y cells. This video shows clover-BAG2 condensates on the microtubules (ruby-Tau) over time. Blend mode (Opacity applied to voxels – 3D impression) were applied using the Imaris software.

File Name: Supplementary Movie 5
Description: BAG2 condensates and Tau following vinblastine treatment in stable SH SY5Y cells. This video shows clover-BAG2 condensates and ruby Tau after vinblastine exposure, a microtubule depolymerizing agent, over time. Vinblastine markedly increased BAG2 condensation.
