# Supplementary material for: Stress routes clients to the proteasome via a BAG2 ubiquitin-independent degradation condensate
Source: Nat Commun. 2022 Jun 2;13:3074. doi: 10.1038/s41467-022-30751-4 (PMC9163039; doi:10.1038/s41467-022-30751-4)
Supplement: Supplementary file 9 — Reporting Summary [file 41467_2022_30751_MOESM9_ESM.pdf]

nature portfolio

Corresponding author(s): Kenneth S. Kosik

Last updated by author(s): May 10, 2022

Reporting Summary

Nature Portfolio wishes to improve the reproducibility of the work that we publish. This form provides structure for consistency and transparency in reporting. For further information on Nature Portfolio policies, see our Editorial Policies and the Editorial Policy Checklist.

Statistics

For all statistical analyses, confirm that the following items are present in the figure legend, table legend, main text, or Methods section.

n/a	Confirmed	
☐	☒	The exact sample size (*n*) for each experimental group/condition, given as a discrete number and unit of measurement
☐	☒	A statement on whether measurements were taken from distinct samples or whether the same sample was measured repeatedly
☐	☒	The statistical test(s) used AND whether they are one- or two-sided<br>*Only common tests should be described solely by name; describe more complex techniques in the Methods section.*
☐	☒	A description of all covariates tested
☐	☒	A description of any assumptions or corrections, such as tests of normality and adjustment for multiple comparisons
☐	☒	A full description of the statistical parameters including central tendency (e.g. means) or other basic estimates (e.g. regression coefficient) AND variation (e.g. standard deviation) or associated estimates of uncertainty (e.g. confidence intervals)
☐	☒	For null hypothesis testing, the test statistic (e.g. *F*, *t*, *r*) with confidence intervals, effect sizes, degrees of freedom and *P* value noted<br>*Give P values as exact values whenever suitable.*
☒	☐	For Bayesian analysis, information on the choice of priors and Markov chain Monte Carlo settings
☒	☐	For hierarchical and complex designs, identification of the appropriate level for tests and full reporting of outcomes
☒	☐	Estimates of effect sizes (e.g. Cohen's *d*, Pearson's *r*), indicating how they were calculated

Our web collection on statistics for biologists contains articles on many of the points above.

Software and code

Policy information about availability of computer code

Data collection	Imaging data (both in vivo and ex vivo) were obtained and acquired using Resonant Scanning Confocal microscopy (Leica SP8) with the corresponding laser wavelength s sequentially sca nned by line when more than one fluorophore was used.<br>Fluorescence correlation spectroscopy (FCS) measurements were performed on a Leica SP8 Resonant Scanning Confocal equipped with a cooled HyD detect or.<br>Flim-Fr et data collection was performed using Fluorescence Lifetime Imaging Measurements (FLIM) on a Leica SP8 microscope equipped with a HyD detect or with a 63 x (1.49NA, oil) objective.<br>Luminescence measurements were performed using a Victor3 plate reader (Perkin Elmer; Waltham, MA). The blots were scanned using a digit al fluorescent imaging system (Odyssey CLX, LI-COR) .<br>Flow Cytometry Protein Interaction Assay (FCPIA): Binding was detected using an Accuri"'C6 flow cytometer.<br>Nucleotide Release Assay: fluorescence polarization was measured (excitation: 485 nm emission: 535 nm) using a SpectraMax MS plate reader.
Data analysis	Images were analyzed in lmaris software (v.9.7.2; Oxford Instruments) and Resonant Scanning Confocal microscopy (Leica SP8). Fluorescence quantification, granule area sum, number and size of condensates, and colocalization were quantified using lmaris software (v.9.7.2; Oxford Instruments).<br>Image- deconvolution algorithms were applied to remove the out-of-focus blur typical for epifluorescence images and improve both lateral and axial resolution. Images were deconvolved with Huygens Essential for Win 64 version 18.10 (Scientific Volume Imaging, the Netherlands, http:svi.nl), using the CMLE algorithm, with SNR: 20 and 40 iterations.<br>FRET was analyzed with the FLIMfit software tool developed at Imperial College London (Version 5.1.1).<br>For FRAP analysis the exponential recovery was determined using easyfrap version 1.11(https://easyfrao.vmnet.upatras.gr/).<br>Fluorescence correlation spectroscopy (FCS): for each set of measurement s, the excitation volume for a specific optical configuration was calibrated using Alexa 488 at (D = 464.23 µm2 s-1) and fitted to a triplet extended 3D model [https://doi.org/10.1038/nprot.2018.040). For determining the average of [BAG2] Dense drop let concentrations, individual cells were masked in Fiji (Version 1.53c) and exported to ilastik

(Version 1.3.3post2) [https://doi.org/10.1038/s41592-019-0582-9] for particle and object selection. After training on a representative set of cells, the entire dataset was processed, and individual droplet identities were exported and analyzed in MATLAB version R2019B.
For Western Blotting data analysis, the blots were quantified with ImageJ (version 1.53k). Graph Pad Prism (v ersion 6.01) was used for statistical analysis.

For manuscripts utilizing custom algorithms or software that are central to the research but not yet described in published literature, software must be made available to editors and reviewers. We strongly encourage code deposition in a community repository (e.g. GitHub). See the Nature Portfolio guidelines for submitting code & software for further information.

Data

Policy information about availability of data

All manuscripts must include a data availability statement. This statement should provide the following information, where applicable:

- Accession codes, unique identifiers, or web links for publicly available datasets
- A description of any restrictions on data availability
- For clinical datasets or third party data, please ensure that the statement adheres to our policy

The data supporting the findings of this study are contained within and provided in the Source Data file

Field-specific reporting

Please select the one below that is the best fit for your research. If you are not sure, read the appropriate sections before making your selection.

☒ Life sciences ☐ Behavioural & social sciences ☐ Ecological, evolutionary & environmental sciences

For a reference copy of the document with all sections, see nature.com/documents/nr-reporting-summary-flat.pdf

Life sciences study design

All studies must disclose on these points even when the disclosure is negative.

Sample size

No statistical methods were used to predetermine sample sizes. Pool cell-based analysis were experimentally done at least three independent times such as WB (Fig 3f, g; Fig 6e, Supp. Fig 3b, Supp. Fig 4b, Supp. Fig 7c, Supp. Fig 10b) and cell viability (Fig 3e, Fig Sb). Single cell-base d analysis were experimentally done at least three independent times in several cells (number described along the work) such as in Fig 1a,b,e-j, Fig 2a,b,c, Fig 3c,d, Fig 4a-c, Fig 6-d, Fig 7; Supp. Fig 1, Supp. Fig 2, Supp. Fig 3a, c, Supp. Fig 5, Supp. Fig 7b, Supp. Fig 9, Supp. Fig 10, Supp. Fig 11). Single cell condensates-based ana lysis were exp erimentall y done at least in three independent times in several condensates per cell (number described along the work) such as in Fig1e,d,j, Fig 2d-f, Fig 4d-f, Fig 5a, Supp. Fig 6b-d, Supp. Fig 7a, Supp. Fig 8).
Sample sizes were chosen to determine whether or not the results were statistically significant. All analysis has shown robust consistency between assays.

Data exclusions

No data was excluded.

Replication

All experiment s were repeated at least 3 independent times. All attempts at replication were successful.

Randomization

Samples, such as wells in pool cell-based analysis, cells in single cell-based analysis and condensates in single cell-based analysis were randomly chosen for imaging acquisition and quantification. Also treatment such as Sucrose, MGl-32, Sodium arsenite, Temperature , LPS, Oligomycin, ML-7243 and Vinblastine were sample randomly chosen.

Blinding

The quantification, whenever possible, was conducted blindly in order to prevent bias in all the single cell condensates-based analysis such as in Fig 1g,i,j; Fig 2c; Fig 3c-d; Fig 7d; Ext. Fig 1b-e (right panels); Supp. Fig 2a-c; Supp. Fig 7b; Supp. Fig 9d.
Immunofluorescence co-localization analysis were done in an automated fashion eliminating human bias such as in Fig 2d-f, Fig 4d-f, Fig 5a, Supp. Fig 6b-d, Supp. Fig 7a, Supp. Fig 8.

Reporting for specific materials, systems and methods

We require information from authors about some types of materials, experimental systems and methods used in many studies. Here, indicate whether each material, system or method listed is relevant to your study. If you are not sure if a list item applies to your research, read the appropriate section before selecting a response.

Materials & experimental systems

n/a	Involved in the study
☐	☒ Antibodies
☐	☒ Eukaryotic cell lines
☒	☐ Palaeontology and archaeology
☒	☐ Animals and other organisms
☒	☐ Human research participants
☒	☐ Clinical data
☒	☐ Dual use research of concern

Methods

n/a	Involved in the study
☒	☐ ChIP-seq
☒	☐ Flow cytometry
☒	☐ MRI-based neuroimaging

Antibodies

Antibodies used	The following antibodies were use d for immunoblottin g (WB) and/or immunofluorescence (IF) :<br>- lnvitrogen, rabbit anti-BAG2 polyclonal antibody [cat # PA5-96794, 1:1000 (WB) and 1:250 (IF)];<br>-StressMarq Bioscience - mouse anti-human HSP70-HSC70 Monoclonal, clone number N27F3-4 [cat # SMC-104, 1:1000 (WB) and 1:50 (IF)];<br>-Novus Biologicals- rabbit anti-proteasome 20S alpha 5 antibody [cat # NBPl -86838, 1:500 (IF)], mouse anti-Proteasome 19S (PA700) antibody [cat # H00005701-M01, 1:50 (IF)];<br>-Santa Cruz, mouse monoclonal anti-CHIP-STUB1 antibody (Cl0) [cat # SC-133083, 1:50 (IF)];<br>-Cell Signaling - rabbit anti-PARP antibody [cat # 9542S, 1:1000 (WB)], rabbit anti-PA28 ⍰ (PSME) and rabbit anti-PA28 ⍰(PSME3) antibodies [cat # 2409 and # 2412, 1:25 (IF)], rabbit anti - K48-linkage Specific Polyubiquitin (D9D5) antibody [cat # 8081S, 1:200 (IF)];<br>-AbCam - mouse monoclonal anti-Tau antibody (Tau-5) [cat # ab805 79, 1:1000 (WB), 1:400 (IF)], recombinant rabbit anti-Dcp1a monoclonal antibody [EPR13822] [cat # EPR13822, 1:500 (IF)], rabbit monoclonal anti -BAG2 antibody [cat# ab79406, 1:2000 (WB, Fig 3g: endogenous);<br>-Provided by P. Davies, Feinstein Institute for Medical Research, Manhasset, NY - mouse anti-paired helical fil ament -1 (PHF-1) monoclonal antibody, and mouse anti-MC1 monoclonal antibody [1:1 000 (WB)];<br>-ThermoFi sher - Phospho-Tau (Ser202, Thr20S) monoclonal antibody (AT8) [cat # MN1020, 1:1000 (WB)], mouse polyclonal anti-PA28γ (SME3) antibody [cat# 89-006-918, 1:100 (IF)];<br>-Sigma-Aldrich - mouse anti-β-actin antibody [cat # A5441, 1:5000 (WB)];<br>-BD Biosciences - mouse anti-p62 lck ligand, [cat #610833, 1:200 (IF)], purified mouse anti-Human G3BP Clone 23/G3BP (RUO) [cat # 611126, 1:250 (IF)];<br>-Enzo Life Sciences - mouse mono-and polyubiquitinylated conjugates monoclonal antibody (UBCJ2) [cat # ENZ-ABS840-0100, 1:200 (IF)];<br>-lnvitrogen - Alexa fluor 488 goat anti rabbit (cat# All00S) or anti mouse (cat# All00l) lgG [1:450 (IF)], Alexa Fluor 555 goat anti- rabbit (cat# A21428) or anti-mouse (cat# A21422) lgG [1:450 (IF)], Alexa Fluor 790 goat anti-rabbit lgG [1:5000 (WB)] and Alexa Fluor 680 goat anti-mouse lgG [1:5000 (WB)].
Validation	-anti-BAG2 antibody (lnvitrogen) was validated by the manufacturer for WB and ICC. https://www.thermofisher.com/antibody/ product/BAG2-Antibody-Polyclonal/PA5-96794. We validated: by WB with a band of expected molecular weight (fused to the fluorophore), by ICC which present similar cellular distribution pattern compared to literature, with the Clover-BAG2 overexpression forming BAG2 condensates as well as its response to osmotic stress. We also validate with its association with Tau on microtubules [https://doi.org/10.1523/JNEUROSCl.4 660-08.2009]<br>-anti-BAG2 antibody (AbCam) [cat# ab79406, 1:2000 (WB, endogenous) was knockout validated by the manufacturer. Was also validated by several citations: https:// www .abcam.com/bag2-antibody-epr3567-ab79406.htm1. We validated the endogenou s staining by WB with a band of expected molecul ar weight and with sgRNA knockdown. We also validated by ICC which present similar cellular distribution pattern compared to the anti -BAG2 antib ody from lnvitrogen.<br>-anti -human HSP70/HSC70 Monoclonal antibody was validated by the manufacturer for WB and ICC and by several citations: https:// www.stressmarq.com/products/antibodies/monoclonal-antibodies/ h sp70-hsc70-antibody-smc-104/?v=7516fd43adaa. We validated by ICC which present similar cellular distribution pattern compared to literature, with the pEGFP-Hsp70 overexpression and its r esponse to osmotic stress and associat ion with BAG2.<br>-anti -CHIP-STUB1 antibody [cat d SC-133083] was validated by the manufactur er for WB and ICC and by several citations: https:// datasheets.scbt.com/ sc- 133083.pdf. We validated by ICC which present similar cellular distribution pattern compared to literature<br>-anti -proteasome 20S alpha 5 antibody was validated by the manufacturer for WB and ICC: https://www.novusbio.com/products/ proteasome-20s-alpha-5-antibodynbpl -86838. We validated by ICC which present similar cellular distribution pattern compared to literature with strong staining in the nucleus.<br>-anti -Proteasome 19S (PA700) antibody [cat # H00005701-M01, 1:50 (IF)] was validated by the manufacturer for WB and ICC: https://www.novusbio.com/products/proteasome-19s-s7-antibody-4c10-2c8h00005701- m01. We validated by ICC which present similar<br>cellular distribution pattern compared to lit erature<br>-anti-PARP antibody was validate d by the manufact urer for WB and by several citations: https:// www.cellsignal.com/products/ primary-antibodies/parp-antibody/9542. We validate d by WB with a band of expected molecular weight as well as two bands of expected molecular weight aft er osmotic treatment due to PARP cleavage.<br>-anti -PA28beta anti-PA28gamma antibody (Cell Signaling) was validated by the manufacturer for WB and ICC and by several citations: https://www.cellsignal.com/products/primary-antibodies/pa28b-antibody/2409 and https://www.ceellsignal.com/ products/primary- antibodies/pa28g-anti body/2412. We validated by ICC which present similar cellular distribution pattern compared to literature.<br>PA28beta is more cytoplasmatic and PA28gamma more nuclear. Also, although PA28-gamma is described as localized to the nucleus, after stress PA28 is r eadily detectable in the cytoplasm [https://doi.org/10.1042/bj200217S8].<br>-anti -PA28gamma antibody (ThermoFisher) were validated by the manufacturer for WB and ICC. https://www.fishersci.com/shop/

products/anti-psme3-polyclonal-abnova/89006918. We validated by ICC which present similar cellular distribution pattern compared to literature. PA28beta is more cytoplasmatic and PA28gamma more nuclear. Also although PA28gamma is described as localized to the nucleus, after stress PA28 is readily detectable in the cytoplasm [https://doi.org/10.1042/bj20021758].
-anti-K48-linkage Specific Polyubiquitin (D9D5) antibody was validated by the manufacturer for WB and ICC and by several citations: https://www.cellsignal.com/products/primary-antibodies/k48-linkage-specific-polyubiquitin-d9d5-rabbit-mab/808l. We validatedby ICC which present similar cellul ar distribution pattern compared to literature with strong staining in the nucleus.
-anti-Tau antibody (Tau-5) was validated by the manufacturer for WB and ICC and by several citations: https://www.abcam.com/tau-antibody-tau-5-bsa-and-azide-free-ab80579.htm1. We validated by WB with a band of expected molecular weight and by ICC which present similar cellular distribution pattern on microtubules compared to literature .
-anti-Dcp1a monoclonal antibody was validated by the manufacturer for WB and ICC and by several citations: https://www.abcam.com/dcp1a-antibody-epr13822-ab183709.html. We validated by ICC which present similar cellular distribution pattern in response to arsenite and/or osmot ic stress compared to literature.
-anti-paired helical filament-1 (PHF-1) monoclonal antibody, and mouse anti-MC1 monoclonal antibody were provided by P. Davies, Feinstein Institute for Medical Research, Manhasset, NY. Those antibodies are well known and validated by WB and ICC in the literature [PHF used by https://doi.org/10.1523/JNEUROSCl.4 660-08.2009 and MC1 used by https://d oi.org/10.1038/srepl1161].
-anti-Phospho-Tau (Ser202, Thr205) monoclonal antibody (ATS) was validated by the manufacturer for WB and ICC and by several citations: https://www.thermofisher.com/antibody/product/Phospho-Tau-Ser202-Thr205-Antibody-clone-AT8-Monoclonal/MN1020. We validated by WB with a band of expected molecular weight.
-mouse anti-βActin antibody was validated by the manufacturer for WB and ICC and by several citations: https ://www .sigmaaldrich.com/US/en/product/sigma/ a5 441?context=product. We validated by WB with a band of expected molecular weight.
-mouse anti-p 62 lck ligand antibody was validated by the manufacturer for WB and ICC and by several citations: https://www.bdbiosciences.com/en-us/products/reagents/microscopy-imaging-reagents/immunofluorescence-reagents/purified- mouse-anti-p62-ick-ligand.610833. We validated by ICC which present similar cellular distribution pattern compared to literature as well as increased granule formation in response to Bafilomycin A1.
-mouse anti-Human G3BP1 antibody was validated by the manufacturer for WB and ICC and by several citations: https://www.biosciences.com/en-us/ products/reagents/microscopy-imaging-reagents/immunofluorescence-reagents/purified-mouse-anti-human-g 3bp.611126. We validated by ICC which present similar cellular distribution pattern compared to literature as well as in creased granule formation in response to arsenite and /or osmotic stress.
-mouse mono-and polyubiquitinylated conjugates monoclonal antibody was validated by the manufacturer for WB and ICC: https://www.enzolifesciences.com/ENZ-ABS840/mono-and-polyubiquitinylated-conjugates-recombinant-monoclonal-antibody-ubcj2/. Replaces Prod. #: BML-PW8810 used by literature (doi: 10.4161/auto.2 9409). We validated by ICC which present similar cellular distribution pattern compared to literature.
All secondary antibodies are well used in the literature with several citations.

Eukaryotic cell lines

Policy information about cell lines

Cell line source(s)	SH-SYSY cells, H4, Hela and COS7 cells were purchased from ATCC.
Authentication	Overexpression and stable cells were authenticated by its correspondent fluorophore and cellular localizat ion and/or by WB. All cells used in the present study were kept in culture following standard methods and authenticated by morphological ana lysis.
Mycoplasma contamination	Cultures were tested regularly for mycoplasma (every 2-3 months). All were confirmed negative.
Commonly misidentified lines (See ICLAC register)	Not used
